# A method for restoring signals and revealing individual macromolecule states in cryo-ET, REST

**DOI:** 10.1038/s41467-023-38539-w

**Published:** 2023-05-22

**Authors:** Haonan Zhang, Yan Li, Yanan Liu, Dongyu Li, Lin Wang, Kai Song, Keyan Bao, Ping Zhu

**Affiliations:** 1 National Laboratory of Biomacromolecules, CAS Center for Excellence in Biomacromolecules, Institute of Biophysics, Chinese Academy of Sciences, Beijing, 100101 China; 2 University of Chinese Academy of Sciences, Beijing, 100049 China

**Keywords:** Cryoelectron tomography, Cryoelectron tomography, Software

## Abstract

Cryo-electron tomography (cryo-ET) is widely used to explore the 3D density of biomacromolecules. However, the heavy noise and missing wedge effect prevent directly visualizing and analyzing the 3D reconstructions. Here, we introduced REST, a deep learning strategy-based method to establish the relationship between low-quality and high-quality density and transfer the knowledge to restore signals in cryo-ET. Test results on the simulated and real cryo-ET datasets show that REST performs well in denoising and compensating the missing wedge information. The application in dynamic nucleosomes, presenting either in the form of individual particles or in the context of cryo-FIB nuclei section, indicates that REST has the capability to reveal different conformations of target macromolecules without subtomogram averaging. Moreover, REST noticeably improves the reliability of particle picking. These advantages enable REST to be a powerful tool for the straightforward interpretation of target macromolecules by visual inspection of the density and of a broad range of other applications in cryo-ET, such as segmentation, particle picking, and subtomogram averaging.

## Abstract (web summary)

Heavy noise and missing wedge effect hamper the efficient visualization and analysis in cryo-ET. Here, authors present a deep learning-based method for directly visualizing and revealing the dynamic states of target molecules.

## Introduction

Cryo-ET has emerged as a powerful method which could record the 3D information of the biological macromolecules; however, many challenges still remain to be addressed^1,2^. First, the noise level of the tomogram is very high due to the radiation sensitivity of the samples, hence the low-dose electron tomography hinders human eyes to identify the features in it^3^. Second, during the data collection, tilt-series images can only be collected within a tilt angular range of approximately ±70° because of the limitation of the specimen holder. This could lead to incomplete 3D information in the Fourier space, resulting in a so-called missing wedge in the tomogram. The effect of the missing wedge is clearly visible in the 3D Fourier transform of the beam direction. The most obvious artefact caused by a missing wedge is the anisotropic resolution, in which objects appear elongated in the direction of the beam axis, i.e., in the Z direction^4^. The EM density in the 3D and 2D slices related to the Z-plane are distorted as a result of this elongation. Therefore, most of 3D segmentation was unable to entail in Z direction and render a highlight extended structure.

To address these challenges in cryo-ET, a variety of methods have been proposed to recover the information and produce high contrast tomograms^5^. During the data collection, dual-axis tomography, in which the tilt series are collected using two perpendicular axes, could be applied^6^. However, this method is limited by the use of a higher electron dose, which may damage the biological specimen^7^. In other studies that have focused on the data processing procedures, a series of algorithms, including the algebraic reconstruction technique (ART)^8^, simultaneous ART (SART)^9^ and simultaneous iterative reconstruction technique (SIRT)^10^, have been proposed to improve the quality of tomograms. These methods, which are mainly based on mathematic calculations, reduce the differences between the calculated projections of the reconstructed tomogram and the tilt series. By using these algorithms, high contrast for visualizing 3D structures can often be achieved from the tomogram. In addition to the above algorithms, the compressed sensing (CS)-based method has also been proven to be effective in recovering the information in electron tomograms^11–13^. It introduces a few priori assumptions in the tomogram, e.g., density positivity and solvent flatness, to constrain the structural features and allow the high-fidelity reconstruction of signals. By applying CS on biological samples, ICON was found to be capable of reconstructing tomograms with high contrast and successfully restoring the missing information^12^. A more recently proposed method, CS-TV^2^, which uses an advanced CS algorithm, could increase the contrast while retaining high-resolution information^13^. However, CS-based methods rely heavily on sufficient signal-to-noise ratio (SNR) and thus require high-contrast tomograms.

In recent years, deep learning algorithms have been increasingly applied in cryo-EM and cryo-ET workflows^14–16^. Learning-based methods, e.g., Topaz-Denoise^17^, have been shown to be advantageous in denoising tomograms. It presents a general 3D denoising model of noise2noise (N2N) for improving tomogram interpretability. In addition to denoising tomograms, deep learning has also been used to recover missing-wedge information. In a recent study, a joint model that was designed to recover the missing-wedge sinogram was proposed^18^. It required a U-Net structure combined with a generative adversarial network (GAN) to reduce the residual artefacts. However, the proposed joint model was still limited to 2D data due to the lack of ground truth for the training network model in cryo-ET. In addition to the aforementioned joint model, an application named IsoNet^19^, which learns from the information scattered in the original tomograms with recurring shapes of molecules based the U-Net framework, is used to recover missing wedges in cryo-ET. However, in the processing of IsoNet, its ground truth still preserves noticeable artefacts with a high noise level. Since the training does not employ the real ground truth of each density directly, the effect of restoring relies on the feature and SNR in the density mask. Actually, the raw tomogram suffers from noise and missing wedge which are irreversible; thus, it makes the acquisition of ground truth very challenging. Therefore, for deep learning strategies aiming at information restoration, it is critical to generate suitable training datasets to train the neural network.

In this work, motivated by the joint model and IsoNet, we proposed a knowledge transfer method for **re**storing the **s**ignal in **t**omograms (REST) to denoise the tomograms and compensate for the missing-wedge information in cryo-ET. To address the issue of the nonexistence of ground truth, two strategies, i.e., subtomogram averaging based strategy (Strategy 1) and simulation-based strategy (Strategy 2), are proposed to generate training pairs. By applying the REST method to different tomogram datasets, we find it is highly robust to noise and performs well in denoising and compensating for the missing wedge effect. Significantly improving the direct visualization of the target macromolecules and their structural dynamics in noisy tomograms, REST can help to identify the target macromolecules in both in vitro and in situ tomograms. Our parallel single particle analysis (SPA) and sub-tomogram averaging (STA) analysis shows that REST-restored densities present highly similar structures to those revealed by the averaging techniques. These results indicate that REST can greatly enhance the visualization of macromolecules and improve the structural interpretability of cryo-ET.

## Results

### Workflow of REST

**Fig. 1:**
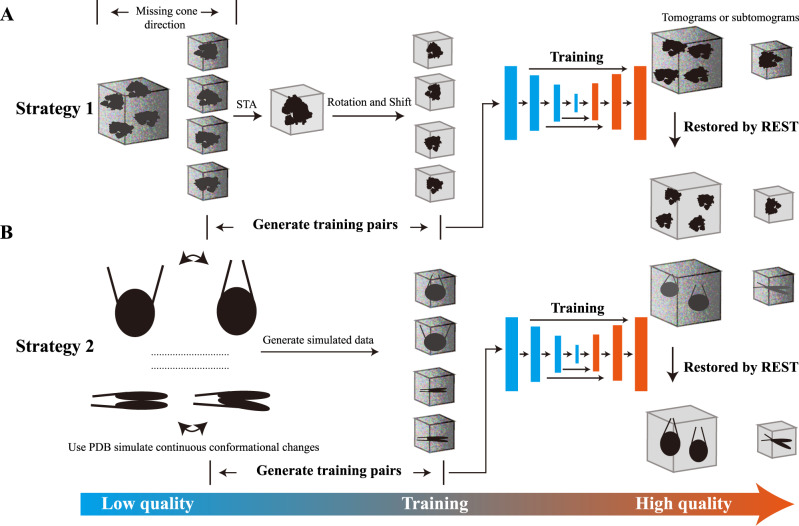
The workflow of the REST method for restoring signal. **A** Strategy 1 consists of generating the training pairs using subtomogram averaging (STA), training the model and restoring information. **B** Strategy 2 consists of generating the training pairs using simulated data, training the model and restoring information. The noise volume images appear distorted. The clean volume images have no distortion with high SNR.

We use U-Net modified from IsoNet^19^, from which the relationship between the input volume (low-quality density) and the ground truth (high-quality density) can be learned, as a model for segmenting dense volume from sparse annotation. The general workflow of REST is comprised of three parts, i.e., generating training pairs, training the model and restoring information, as depicted in Fig. 1. A detailed guideline and tutorial of REST can be found at the GitHub [https://github.com/Zhang-hn1125/REST].

### Generating training pairs

In REST, two strategies are used to generate training pairs. In Strategy 1, subtomogram averaging (STA) density map of the target object (high-quality density) and its corresponding raw particles (low-quality density) are used. In Strategy 2, density map generated from PDB structure (high-quality density) and its corresponding simulated particles with imposed noise and flexibility (low-quality density) are used.

**Strategy 1: Subtomogram averaging based strategy**

Step 1: Subtomogram averaging

Subtomogram averaging was first performed by using a routine STA technique with limited amounts of particles. The generated CTF and missing wedge corrected averaging map with higher SNR was then used as the ground truth for the training pairs established between the individual raw particle and the STA density in the orientation corresponding to that raw particle. Here, assigning each raw particle a relatively accurate orientation parameter is critical for establishing an effective training pairs. The accurate alignment parameters for each particle could be used to efficiently reduce the loss in training.

Step 2: Extraction of subtomograms

The raw subtomograms that participated in the averaging in step 1 were extracted as the input of the training data.

Step 3: Generation of the ground truth

According to the alignment parameters from each subtomogram, the averaged map was rotated and shifted to generate the ground truth. By coupling the input of training data from step 2 and the ground truth, the training pairs were obtained.

**Strategy 2: Simulation-based strategy**

Step 1: Generation of dynamic models using normal mode analysis (NMA)

When a structure of the target object is available, Strategy 2 is recommended. Here, Normal mode analysis (NMA), a method for molecular mechanics simulation^20^, was first used to generate a series of dynamic conformational changing models from the static (pseudo) atomic model of target molecules. Based on the prior knowledge of the target molecule, the conformations of interest were selected among all of the dynamic models for the next steps.

Step 2: Generation of the ground truth

The selected dynamic models were converted to EM density, rotated and shifted in the 3D space using random Euler angles and shifts, and taken as the ground truth of the training pairs.

Step 3: Generation of the simulated data

The projection images covering different tilt angle ranges were generated using the EM density (ground truth) in step 2. The projection images were superimposed with different degree of noise to adjust the SNR and modulated with the contrast transfer function (CTF). The tomogram volume was then reconstructed from the modulated projection images and taken as the input of the training data. Details of the method used to simulate the data are provided in the Methods section. By coupling the input of the training data and the ground truth from step 2, the training pairs were obtained.

Typically, Strategy 1 is useful in the situation that an STA averaged map can be obtained for the target object, while Strategy 2 is more useful when a structure is available for the target object. The later strategy also has the potential to reveal the structure variations of target object by using NMA and/or other methods to simulate the structural changes.

### Training the model

We employed a U-Net-based voxel-wise network derived from IsoNet. One of the advantages of U-Net is its ability to segment the dense volume from sparse annotation^21^. Therefore, U-Net is particularly suitable for segmenting parse features from cryo-electron tomograms containing heavy noise and elongated artefacts due to the missing wedge effect. The main blocks in U-Net are built from stacking multiple layers, which are used for 3D convolution and deconvolution. The convolution and deconvolution layers are used for extracting the features of target objects and recovering the high-resolution features. After training, the mapping relationship is established between the low-quality particles and the ground truth (i.e., the high-quality particles) with their corresponding orientations. The mapping relationship and knowledge learned from the training pairs can then be transferred to restore the low-quality real density.

### Restoring information

To evaluate the robustness of REST when it is influenced by missing information and noise, we tested the restoring capability of REST using a series of datasets, including the simulated tomograms under different conditions (SIM1, 2, 3, 4). Interestingly, we found that REST could handle the disturbances of noise and missing wedges well, and good performance was achieved in restoring information even when the SNR was reduced to 0.01 and the tilting range covering only −40° ~40°. The correlation coefficient (CC) between the prediction and the ground truth was close to 1.0 (Supplementary Fig. 1), suggesting an almost complete restoration from the noisy volume in the predicted particle. Notably, the input volume for restoration does not require preprocessing steps such as deconvolution or filtering to improve the SNR, that is, the raw reconstructed tomogram from WBP could be used as the input directly. Using the raw WBP reconstructed tomograms as the input has an advantage when retaining the high-resolution features, as most of the preprocessing or denoising steps in tomograms, such as low-pass filtering, removes these high-resolution signals and results in an adverse influence on restoration.

### REST shows the capability to enhance SNR and reduce resolution anisotropy in real data

**Fig. 2:**
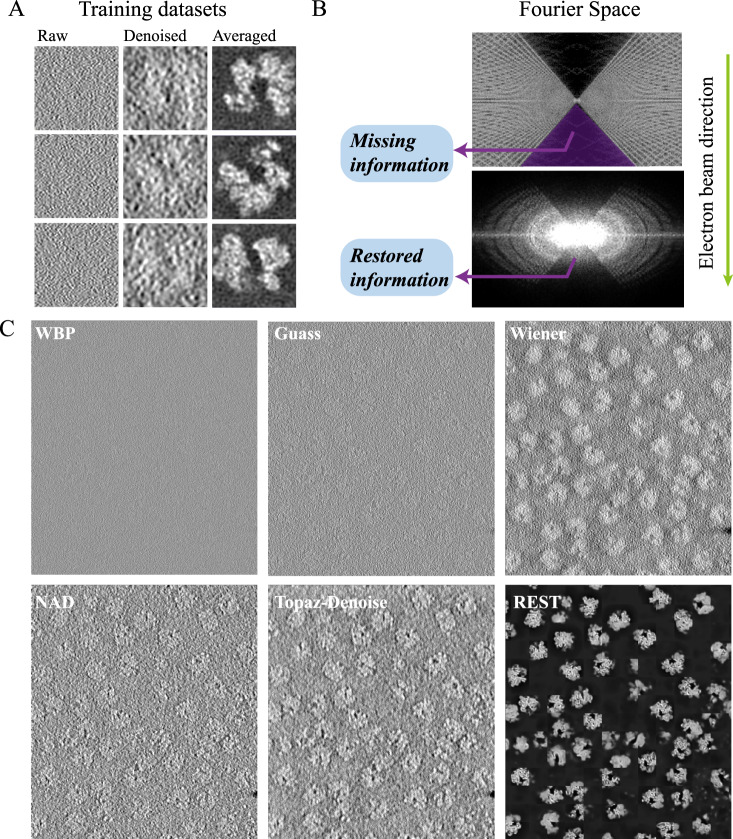
Results from the real datasets of ribosomes (EMPIAR−10045) using REST. **A** Examples of three training pairs. Left: the raw particles extracted from the tomograms (input); Middle: the denoised particles (filtered by Wiener filter), which are easier to observe; Right: the averaged map that rotated and shifted based on the alignment parameters. **B** Fourier transforms of the raw data and restored tomogram. **C** Comparison of the raw data (WBP) and the denoised data using the Gaussian filter, Wiener filter, NAD, Topaz-Denoise and REST methods. The REST method could significantly remove noise that appears in the tomographic slices.

To evaluate the restoration capability of REST on the (sub)tomograms after training, we first applied it to the EMPIAR-10045 dataset (EM1), which contains the tomograms of ribosomes (~25 nm), a stable sample with high abundance in vivo. For this dataset, strategy 1, i.e., the subtomogram averaging (STA)-based strategy, was used to generate the training pairs. We directly extracted the particles from the raw tomograms as input, calculated an STA averaged map and rotated and shifted the average map corresponding to the orientation of each particle as the ground truth (Fig. 2A). After training the model and restoring information using REST, we found that the corresponding missing-wedge information was significantly recovered in the Fourier space (Fig. 2B) and the restored tomograms were highly visible (Fig. 2C).

Compared with other denoising methods, such as the Gaussian filter, Wiener filter, Topaz-Denoise, nonlinear anisotropic diffusion (NAD), we found that REST achieved a stronger noise removal performance and reserved more signals in the 2D slices (Fig. 2C). We quantitatively assessed the denoising performance by measuring the SNR of raw slices and slices denoised with these methods. Since the ground truth was not available for the datasets, the SNR was estimated in a similar approached that in Topaz-Denoise^17^. First, we averaged ten slices into one micrograph. Then, we selected 10 paired signal and background regions across the micrographs. Given the signal N and background pairs ${x}_{s}^{i},\, {x}_{b}^{i}$, the mean and variance of each background region is ${\mu }_{b}^{i},\, {v}_{b}^{i}$. We defined the signal for each region as ${s}^{i}={x}_{s}^{i}-{\mu }_{b}^{i}$ and calculated the mean and variance of the signal region, ${\mu }_{s}^{i},{v}_{s}^{i}$. The average SNR in dB for the regions is defined as:

1 $${{{{{\rm{SNR}}}}}}=\frac{10}{N}\mathop{\sum }\limits_{i=1}^{N}{{{\log }}}_{10}({v}_{s}^{i})-{{{\log }}}_{10}({v}_{b}^{i})$$

As shown in Supplementary Table 1, the SNR was improved by approximately 0.5 dB over the raw micrographs when using the conventional methods. Notably, the SNR was improved by 7 dB over the raw slices and approximately 6 dB over other methods when using REST, which indicates that a significant improvement in SNR enhancement is achieved.

**Fig. 3:**
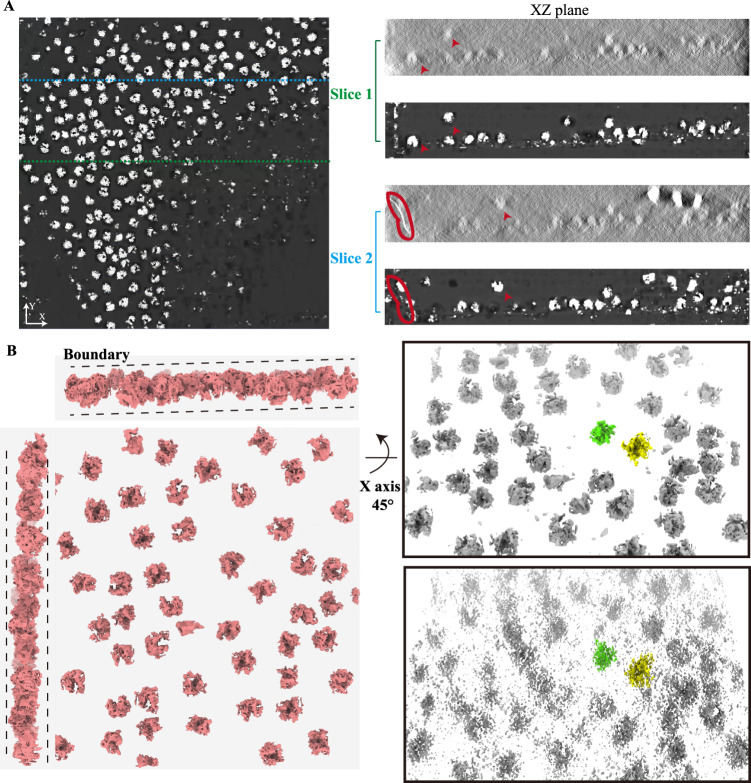
REST enabled the restoration of 3D density and the accurate identification of particles in all directions. **A** Left: Views of the XY-slice of the tomogram restored by REST; Right: Two XZ-slice views of conventionally denoised (Wiener filter) tomogram (top) and REST restored tomogram (bottom). The red arrows indicated the corresponding ribosomes (target signal). The red ellipse indicated the edge of the carbon film (non-target signal). **B** Left: A 3D rendering of the tomogram restored by REST. Right: The tomogram restored by REST (top) had a better restoration capability than that in the conventionally denoised tomogram (Wiener filtering) (bottom). Both the tomograms were rotated around the x-axis by 45° corresponding to left panel.

Additionally, after restoration by REST, we found that each particle could be identified clearly not only in the XY-plane but also in the XZ-plane, with few elongation artefacts (Fig. 3A). Thus, REST enables the accurate identification of particles in all directions. In addition to the 2D slices, compared with the density processed by Wiener filtering (in order to visualize the density), REST was able to restore the 3D density of the particle (e.g., the green and yellow particles) with almost no visible elongation and distortion (Fig. 3B). However, it was worth noting that REST could perform good restoration only toward the target molecules. For other non-trained objects in the tomogram (such as those of carbon films in the tomogram, Fig. 3A), the restoring would not be accurate.

In addition to strategy 1, strategy 2, i.e., the simulation-based strategy, was also tested. This strategy was applied to the dataset of nucleosomes (~10 nm) that we reconstituted (EM2). The reason we selected nucleosomes is that nucleosomes have area notably smaller size than the ribosomes studied above, and are known to be highly dynamic (see below). A similar improvement in terms of compensating for the missing wedge and denoising the tomogram was noted on the nucleosome dataset (Supplementary Table 1, Supplementary Fig. 2). These results suggest that both strategy 1 and strategy 2 could be implemented to achieve an enhanced SNR and reduced resolution anisotropy and could be successfully applied to real tomograms.

### Application of REST to simulated flexible sample revealed conformational changes in the individual particles

**Fig. 4:**
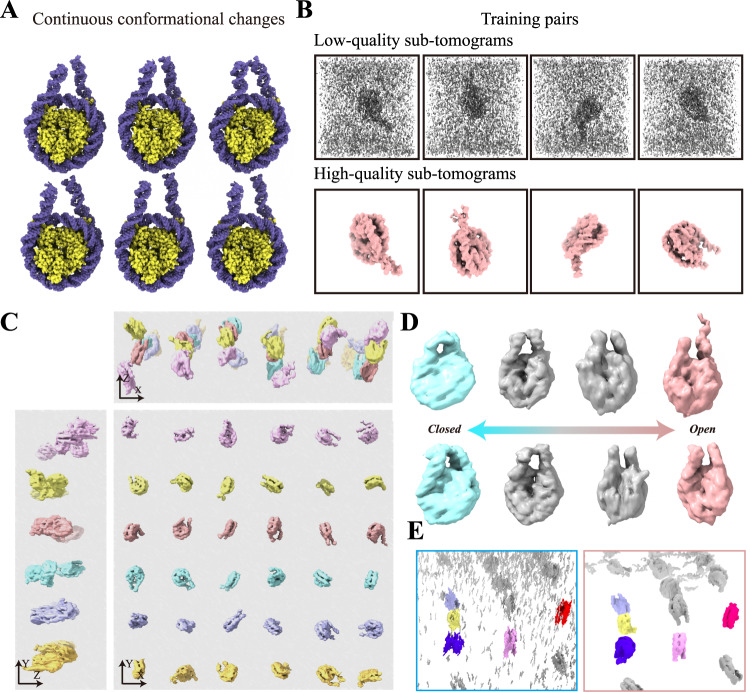
REST revealed continuous conformational changes of nucleosome linker DNA in simulated dataset (SIM5). **A** A series of dynamic nucleosomes generated by using NMA. **B** Examples of four training pairs. Top: the input of training pairs generated from the corresponding ground truth particle with noise (SNR 0.1) and a missing wedge (±40°) superimposed (low-quality). Bottom: the ground truth of the training pairs generated from the atomic model (high-quality). **C** A 3D rendering of the restored tomogram. Each nucleosome could be identified with different linker DNA conformation. **D** Eight representative particles from **A** showing the motion of linker DNA from closed state (cyan) to open state (pink) via middle transitions (gray). **E** Compared with the 3D density improved by Topaz-Denoise and IsoNet (left), REST could effectively clean the noise density and eliminate elongation and distortion (right). The region was from simulated tomogram of particles in **A** and rotated around the x-axis by 45°.

It is known that most macromolecules are not strictly rigid but are flexible entities with continuous conformational transitions when performing their biological functions^22,23^. Although the STA method can be used for classification to study different conformations, particles with continuous conformations in the subtomograms are rarely assigned to the same class^24,25^. In addition, the number of particles in each class is typically insufficient to obtain a high-quality averaged result. Thus, the complicated cell environment and continuous conformational changes of the specimen make disentangling the data heterogeneity by STA difficult. Here, by using NMA, we generated a simulated dataset of 177 bp nucleosomes (SIM5) that have flexible flanking linker DNA and continuous conformational changes as a test object (Fig. 4A). The training pairs were obtained using strategy 2 as shown in Fig. 4B. After training, the test densities restored by REST were highly consistent with those of the ground truth (Supplementary Fig. 3).

Remarkably, using strategy 2, we found that REST could be also used to discern a series of conformational changes in the tomogram. For example, in the simulated tomogram of dynamic nucleosomes dataset (SIM5), the REST-restored tomogram was highly consistent with the ground truth, and the contrast was significantly improved compared with the raw data. In the Fourier space, the missing wedge is also compensated well (Supplementary Fig. 3). In addition to the 2D slices, REST can also restored the 3D density, while the structural variation, e.g., the linker DNA breathing motions between the open and closed states^26^, could be clearly visualized and unambiguously identified (Fig. 4C, D). By analysing the missing-wedge information, we found that REST could effectively eliminate elongation and distortion, which showed a significant improvement compared with IsoNet (Fig. 4E). These results suggested that REST could be used to directly identify and display the conformational changes of the dynamic structures.

Interestingly, in REST, the data for training does not necessarily include all the possible conformations of the particles. In this study, we only used limited amounts of conformations generated by strategy 2 to train the network. Nevertheless, many conformations that were not included in the training dataset could still be identified. This result indicates that REST could transfer knowledge from limited prior information to analogous information of a broad cast.

### Applying REST to real nucleosome data with different lengths of DNA reveals the individual characteristics of the particles

**Fig. 5:**
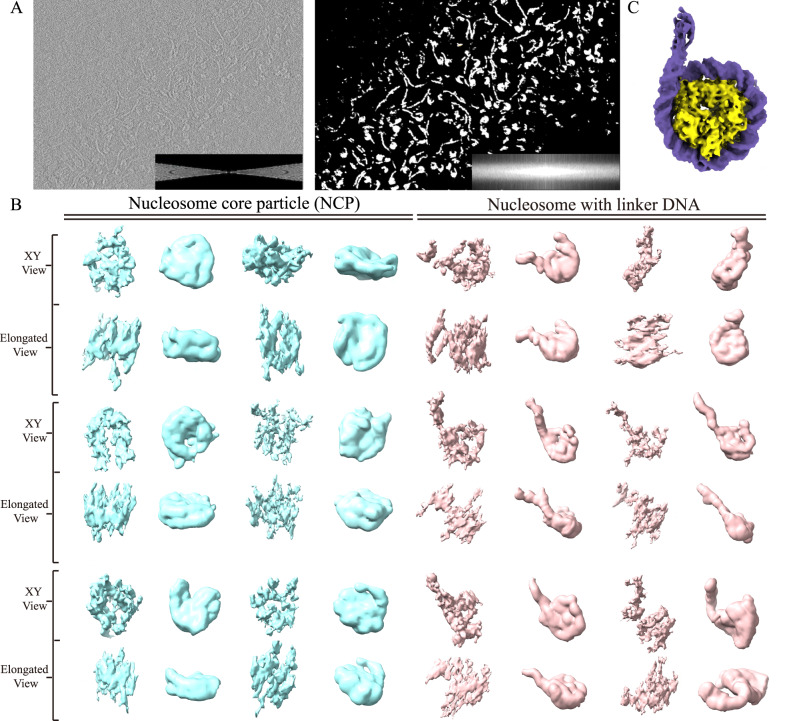
REST could directly reveal versatile characteristics of dynamic nucleosomes. **A** The 2D slices of the raw tomogram (left) and REST-restored tomogram (right). The Fourier transforms are shown in the right corners. **B** Comparison of volumes denoised by the combination of Topaz-Denoise and IsoNet (T-I density, left) and REST-restored density (right). For clarity, each nucleosome core particle (cyan) or nucleosome with linker DNA (pink) contains two different views: the XY-view and the tilted view with the elongation (elongated view). **C** The 3D map of the reconstituted nucleosome with linker DNA by conventional single-particle reconstruction shows the asymmetric linker DNA.

In addition to the simulated nucleosome dataset, real tomograms of nucleosome samples with linker DNA were also tested. We reconstituted nucleosome with linker DNA particles, mixed them with nucleosome core particles (NCP) without linker DNA as a control, and collected a series of electron tomography datasets (EM3) for testing REST. According to the result, we found REST also presented a notable improvement in SNR and recovering the missing wedge in Fourier space (Fig. 5A). The restored tomogram was shown in Supplementary Fig. 4. We used the combination of Topaz-Denoise and IsoNet to denoise and compensate for the missing wedge in tomogram, which is referred to as the T-I density hereafter. Compared with T-I density, the 3D density of each subtomogram after REST restoration was less elongated and distorted, and thus, closer to the real structure (Fig. 5B). We also statistically analysed the CC value between the density restored by REST and the T-I density (Supplementary Table 4). The high CC value achieved by REST indicated that the restored densities were authentically derived from the raw tomogram. As a consequence, nucleosomes with linker DNA could be readily distinguished from NCP by visualizing the flanking linker DNA out of the nucleosome (Fig. 5B). Compared with the wrapped DNA on the NCP, the extra unwrapped linker DNA was apparently flexible, and thus, showed versatile conformations. By applying REST to the nucleosomes with flanking linker DNA, we could distinguish the symmetric and asymmetric linker DNAs with extended or curved conformations that coexist within the nucleosomes (Fig. 5B). This kind of structural flexibility is consistent with the nucleosome variations in interphase and metaphase chromosomes^27^. These results indicated that interpretable information, such as dynamically changing nucleosomes with different conformations, could be directly derived from the elongated and noisy subtomogram after restoration by REST.

In addition to the REST method, we also applied conventional single particle analysis (SPA) to reveal the 3D structure of reconstituted nucleosomes with linker DNA. Subjecting approximately 200,000 particles to averaging, the structure was resolved at a resolution of 3.7 Å, which showed an asymmetric linker DNA at one end of the nucleosome (Fig. 5C). Interestingly, this specific conformation, as shown in the structure resolved at high resolution by SPA, was also found in the architectures discerned by REST without averaging. Therefore, the results indicated that REST could directly reveal versatile characteristics of target macromolecules, thus had a great practical application in cryo-ET.

### Application of REST in lamellae of frog erythrocyte nuclei improved the interpretation of nucleosome

**Fig. 6:**
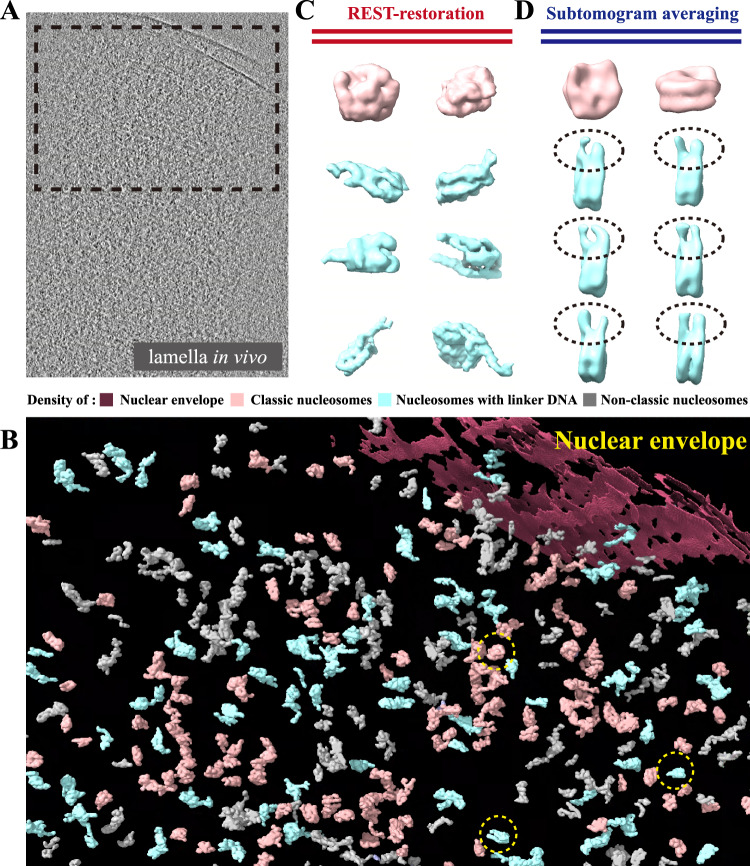
REST could directly improve the interpretation of nucleosome in vivo. **A** Tomographic slice of frog erythrocyte nuclei thinned by cryo-FIB. **B** 3D REST-restored view of the tomographic data in **A**. Yellow circle labelled the particle displayed in **C**. **C** The densities of nucleosome which were directly extracted from REST-restored tomogram. **D** The different conformation of subtomogram averaged map calculated from the same sample. Nucleosomes without (pink) or with linker DNA (cyan), and other non-classic nucleosome-like densities (grey) are coloured differently in **B**–**D**.

Besides the above purified and reconstituted samples in vitro, we also applied REST to the cryo-ET of lamella produced by cryo-FIB, which represented the molecular structures in vivo. The lamella often has a dominant thickness of ~150–200 nm which is much thicker than the molecular layer on the grid (usually <50 nm). Thus, the cryo-ET of lamella has a heavier background noise due to multi-layered samples and complexed cell environment. Therefore, the interpretation of cryo-ET of lamella is typically much more challenging. In this study, we acquired the 3D cryo-ET of lamellae of frog erythrocyte nuclei prepared by cryo-FIB and applied REST to them (Fig. 6A).

Interestingly, the REST-restored tomograms clearly rendered nucleosome densities dispersed inside of nuclear envelope (Fig. 6B). Different from the reconstituted nucleosomes, the nucleosomes in situ had heterogeneous composition including different DNA sequence, histone modifications, histone variants, etc. These differences could lead to the irregular structure of nucleosome. In the REST-restored tomogram, we found that most of densities presented a clear feature of classic nucleosome structure (Fig. 6B, C, pink), while some of them were less obvious but still with nucleosome-like features (Fig. 6B, grey). Interestingly, we found that some densities could be clearly recognized as nucleosomes with linker DNA, which presented variant conformations after REST restoration (Fig. 6B, C, cyan).

In addition to the REST restoration, we also applied the averaging-based STA method to reveal the 3D structure of nucleosomes in the same tomogram of frog erythrocyte nuclei (Supplementary Fig. 5). The averaged results also showed a similar structure of classic nucleosome (Fig. 6D, pink) and the nucleosome with flexible linker DNA (Fig. 6D, cyan). These results indicated that the REST-restored densities could render fundamental features which were compatible with the averaged map and help the structural interpretation of target macrobiomolecules in vivo.

### Restoration by REST facilitated particle picking and orientation determination of subtomogrms in STA

To free researchers from particle picking work on tomograms, a number of methods have been proposed^15,16^. Usually, the template matching method is the first choice if a template is available. However, this method suffers from missing wedges and noise; thus, the calculated CC value between the subtomogram and template is relatively low. Consequently, false-positive hits and unreliable results often occur. Since the REST method can be used to achieve both enhanced SNR and missing wedge compensation, it can also be used as a preprocessing method in particle picking before template matching. We tested both simulated data and the corresponding REST-restored data for template matching. The statistical offset from the ground truth centre and the CC value between the subtomogram and template were compared to evaluate the performance of REST restoration on picking particles.

**Fig. 7:**
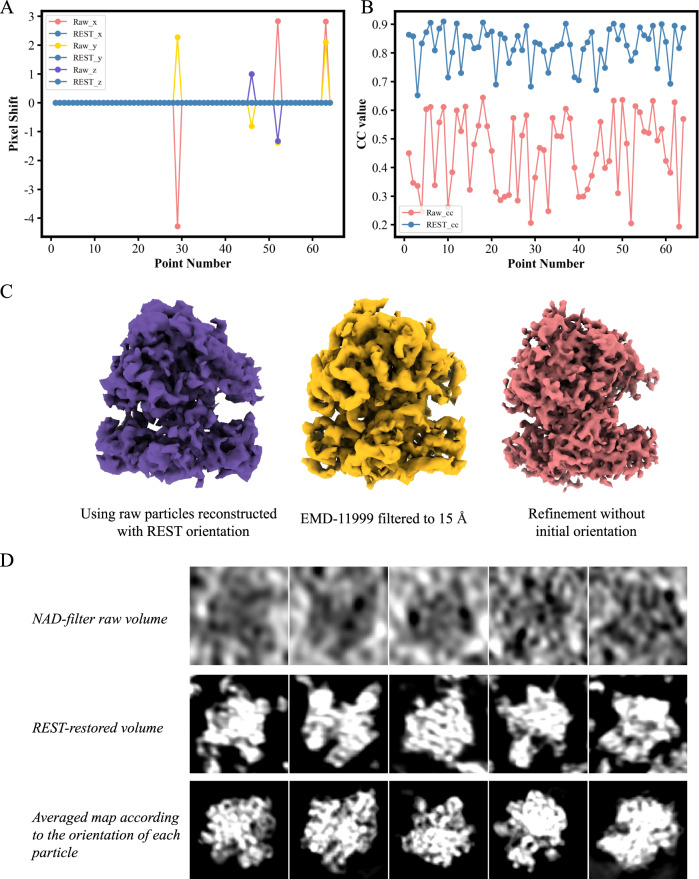
REST could facilitate particle picking and assist the determination of orientation in STA. **A** Comparison of particle picking accuracy in the raw tomogram and in the REST-restored tomogram. The offsets of each picked particle in the X-, Y-, and Z-directions to the ground truth either in the raw tomogram or in the REST-restored tomogram are shown. **B** Comparison of the CC values calculated between the picked particles and templates in the raw tomograms and in the REST restored tomograms, respectively. **C** REST-restored particles present roughly accurate orientations. Left: Direct reconstruction result of the 892 raw particles using the orientation calculated from REST-restored density, Middle: The averaged map calculated from the same raw particle set using the traditional STA strategy. Right: EMD-11999 filtered to 15 Å for comparison. **D** Top: Raw subtomograms (contrast enhanced by NAD filtering). Middle: Subtomograms restored by REST. Bottom: Remapped averaging density map in the position of raw particle corresponding to the orientations determined by STA.

As shown in Fig. 7A, the coordinates calculated from the REST restored tomograms are extremely consistent with the ground truth centre. In contrast, the coordinates calculated directly from the raw tomogram present variant deviations, although most centres of particles are identified right. However, as shown in Fig. 7B, the CC values calculated from the two tomograms are noticeably different. The CC value, which reflects the confidence of the particle centre and the orientation, was significantly improved in the tomogram restored by REST. This was most likely contributed to the high consistency between the restored density and the real signal. These results indicate that using tomograms restored by REST could greatly improve the reliability of particle picking with a very high CC value (Fig. 7A, B).

To validate the accuracy of the orientation calculated by REST-restored particles, we used the in situ ribosome data from pneumococcus^28^ (EMPIAR-10499) as an example (Fig. 7C). We manually picked 892 particles and calculated the orientation parameters of each REST-restored particle using the averaged map EMD-11999^28^ as reference. The orientation parameters obtained in the last step were then directly applied to the raw particles for direct reconstruction (Fig. 7C, purple). The reconstruction is compared with the filtering results of EMD-11999 (Fig. 7C yellow). We found that they are highly consistent. These results suggest that the orientations of REST restored particles, including those particles in situ, can be accurately calculated, and the parameters can be directly used to assist STA process (Supplementary Fig. 6). Meanwhile, we also made a reverse evaluation. All of the picked raw particles were first subjected to a conventional STA process without an initial orientation assigned. The averaged map (Fig. 7C red) was then mapped back to the position of each raw particle, which reflects the ‘ground truth’ of the particle. The mapped back averaged map of each particle is then compared with that of REST-restored one, which shows that they are highly consistent in terms of particle shape and orientation (Fig. 7D). These results suggest a reliable restoration by REST. Meanwhile, it also indicates that the restoring parameters are accurate which could assist the process of STA to obtain the right orientations of particles quickly. That is, the REST-restoration method has the potential to facilitate the STA process by taking advantage of the REST-restored subtomograms containing higher SNR and relatively accurate initial orientations which can be used in the substantial steps (Supplementary Fig. 6).

## Discussion

Cryo-ET has been increasingly used in 3D structural studies of native biological samples^29–31^. By reading reconstructed tomograms, biomacromolecule information, including its spatial arrangement, architecture, or even specific orientation, is expected. However, due to both the low SNR and missing wedge effect, the interpretation of the tomogram is largely limited. Sometimes even the identification or classification of the target biomacromolecule is notoriously difficult. To overcome this drawback, subtomogram averaging, which is laborious and challenging, has often been necessary. In addition, this kind of averaging method usually requires disentangling the continuous architecture from the flexible samples. In contrast, the REST method presented in this study could be used to significantly enhance the SNR and reduce resolution anisotropy without averaging. This could greatly help to reveal the individual characteristics of each biomacromolecule. The rendered architecture also produces a clean density that is sufficient enough for us to distinguish fundamental features, thus making the (sub)tomogram directly interpretable.

It is well known that deep learning relies heavily on training datasets. Thus, using real data with the ground truth for training is the best choice to ensure the network performance. However, the raw tomogram suffers from noise and an irreversible missing wedge, making the acquisition of the ground truth very challenging. STA provides an alternative approach to obtain the ground truth of the raw data. This approach, Strategy 1, could be used to consequently establish a mapping relationship between the raw data and an averaged map for restoration. Nevertheless, as most macromolecules are flexible, the averaged structures present insufficient features, and thus, a valid mapping relationship is rarely established. To address this situation, in this study, we found an alternative strategy, Strategy 2, that conversely degrades high-quality data to simulate low-quality data that are close to the raw data and establish a relationship between them. As long as the neural network can learn the mapping relationship well, the model can be migrated into raw data to generate the corresponding high-quality data. Apparently, the most challenging issue is how to make the simulated data closely related to the raw data. We found that the analogous contrast and elongation are the keys to simulating subtomograms in cryo-ET. Interestingly, introducing the conformational changes in training datasets would enable REST learn more knowledge of flexibility and greatly improve the restoration ability. This step could also make the simulated data look closer to real data and greatly help the identification of polymorphic structures of macromolecules, especially in vivo. To our experience, it is worth noting that REST method can only reliably capture the analogous variability presenting in the training data generated by NMA or other structure dynamics simulation methods. It would not be able to capture drastic changes, which are likely not well learned and transferred by the current network.

It is also worth noting that REST is based on the U-Net framework, which contains complex layers (Supplementary Fig. 7A). During the restoration, REST goes through a process from downsampling for extracting the features to upsampling for recovering the information, which could be considered as a nested non-linear filter that transited the input to the output based on the established training model (Supplementary Fig. 7B, C). Therefore, the restoring process is completely different from the matching or replacement process which is usually subject to a searching or scoring calculation. In REST, like in the other deep learning strategies, after a model has been trained with limited number of training pairs, the knowledge can be transferred and applied to other similar objects.

In most segmentation methods, e.g., EMAN^32^, the features are manually or automatically labelled in 2D slices. In the REST method, the receptive field is boarded to 3D. The perceptron synchronously globally to locally learns the relationship of the two maps in 3D; thus, the model could eliminate the artefact found in 3D. Moreover, most of the methods for segmentation are highly sensitive to the SNR. Interestingly, in REST, the preprocessing of the input dataset is not needed, i.e., the raw tomograms from WBP could be directly used for restoration.

Attributed to the improved visualization of the 3D densities, REST could be used in the following situations: 1) when the structure of the target molecule is known (either from PDB, SPA, STA or Alpha Fold prediction), REST could be used to directly achieve a significantly improved visualization of target macromolecules and/or identify the contextual information (conformation, orientation, spatial distribution, etc.); 2) if the training pair datasets of target molecules could be established by either STA (strategy 1), or using known structures from PDB (strategy 2), REST could be used to extend the dataset by showing where the target particles locate in the (other) tomogram(s), which could greatly facilitate the particle picking; 3) when there is a requirement of analyzing the heterogeneity of the target particles or structural dynamics, REST could be used to disentangle the data heterogeneity and reveal continuous conformational changes of the specimen, which might not be resolved by averaged-based methods; 4) REST could be applied to other cryo-ET tasks such as assisting in the determination of orientation in STA.

Compared to the sub-tomogram averaging (STA), REST presents several noticeable benefits: 1) Once the model of the target was established, it could be used for nearly all the tomograms which contain the target particle; 2) Various particle states, even the continuously changing conformations, could be directly revealed without the classification process; 3) The restored particles by REST could be displayed with accurate conformation, orientation, and spatial distribution without the remapping process.

As mentioned above, REST can be used to reveal each particle state without the need for time-consuming STA. However, it is worth noting that REST cannot retain the high-resolution information from raw data. This is because the process of training, which is essentially a regression problem, needs to reduce the error between the ground truth and restored volume. In practice, the low-frequency information accounts for the majority of the signal, whereas the high-frequency signal is under the noise and hardly to be labelled accurately. Incorrect results will backproject and update the weight in network. This process leads the loss of high-frequency information in order to converge the loss function. Nevertheless, in many situations, the restored density is sufficient for the distinction of the shape and other fundamental features, as shown in the above examples.

In addition to the loss of high-frequency information, there are also potential limitations of the method: 1) REST is only able to reliably restore known objects because the establishment of training pairs required a persuasive ground truth whose structure must be obtained in prior; 2) REST mainly shows restoring effect toward its trained target. For untrained structures or non-target signals in the tomogram, the restoration would not be accurate; 3) REST restoration would generate some discontinuous densities which could be removed by simply a command like “hiding dust” in most cases, but users should be aware and look at the raw data when interpreting resulting structures in REST-restored tomograms.

If there are two or more targets of interest, e.g., ribosome and HBV viral capsid (~3.8 MDa and ~30 nm in diameter), to be restored, one could use REST to establish a separate model for each target, restore each of the targets respectively, and combine them together (Supplementary Fig. 8). When the HBV capsid structure^33^ (EMD-20670) was used as the training model, a ball-like density very similar to the HBV capsid structure can be well restored (blue arrow in Supplementary Fig. 8A and pink density in Supplementary Fig. 8C), while the other restored densities appear mostly junk-like and discontinuous (Supplementary Fig. 8A, yellow arrow) which can be removed by a ‘hide dust’ operation. Vice versa, when the ribosome structure^34^ (PDB: 4V8M) was used as the training model, a lot of ribosome-like densities (cyan, Supplementary Fig. 8C) can be seen in the REST restored tomogram. The separately restored tomograms can then be combined to get the restored densities with two or more target structures (Supplementary Fig. 8B). Further study, for example, establishing a multi-targets oriented network which could be used to train multiple samples simultaneously, would be necessary to better deal with the situation.

In conclusion, REST presented in this study provides a way to enable the direct observation of fundamental architectures and conformational changes for functional interpretation without the laborious and challenging averaging process. Thus, it could be of broad utility to the cryo-ET community by the function of restoring a clear signal like picking particles in a noisy background, segmenting the target feature, identifying dynamic or flexible architectures, obtaining the density without elongation as the initial reference for STA and even guiding the particles to be classified and aligned for STA.

## Methods

### Implementation specifics of the REST method

In strategy 1, to process the ribosome data used in this study, the STA steps were followed according to the protocols in Relion^35^. Five tomograms in EMPAIR-10045 were used to perform STA while 3006 particles were used for averaging. The 2578 averaged subtomograms were first extracted using Relion^36^ and then used as the input to the training data. According to alignment parameters in star files, *e2proc3d.py*^37^ was used to rotate and shift the averaged map to generate the ground truth. By combining the raw particles and the averaged maps, which were reset to the corresponding orientation, the training pairs were obtained.

In strategy 2, NMA was implemented in the target object. According to the prior of the target model, a series of atomic models were generated by NMA in HEMNMA-3D^24^, in which possible conformations were selected. The atomic model was converted to the EM density using *e2pdb2mrc.py*^37^, and the density was then rotated and shifted in the 3D space using random Euler angles and random x, y, z shifts as the ground truth. The simulated data and training pairs were then generated.

### Simulating datasets based on experimental parameters

To generate the simulated dataset for each subtomogram or tomogram, the following steps were performed:

- Rotate the obtained averaged map or the density from the atom model at random Euler angles, with random x, y, and z displacements (*e2proc3d.py*). For example, by using the density converted from the atom model, 3000 subtomograms (64 voxels, 4.4 angstroms) were generated.
- Project the ground truth according to the corresponding collection conditions (e.g., ±60°, 2°) by using the *relion_project* toolbox.
- Perform CTF modulation on each projection. Gaussian noise is added by using *xmipp_phantom_simulate_microscope*, and then the CTF phase is inverted.
- Reconstruct the tilt series using *relion_reconstruct* to obtain the simulated data with missing wedges and noise.

### Preparation of test samples and cryo-vitrification

For the recombinant nucleosomes, the histone octamers were reconstituted as previously described^38^. Briefly, the H2A, H2B, H3 and H4 were mixed at the equimolar amounts in unfolding buffer (7 M guanidinium HCl, 20 mM Tris HCl, pH7.5, 5 mM 2-mercaptoethanol), and then dialyzed in refolding buffer (2 M NaCl, 10 mM Tris HCl, pH 7.5, 1 mM EDTA, 5 mM 2-mercaptoethanol). The resulting histone octamers were purified through a size exclusion chromatography column (Superdex 200, GE Healthcare), and the peak fractions were collected and stored. We used 147 bp and 177 bp 601 DNA to reconstitute nucleosome which was performed as described. Then we mixed 147 bp and 177 bp nucleosome core particles in an 1:1 molar ratio for freezing. For cryo-EM analysis, 3 µl of sample was applied to Quantifoil R2/1 Au 300 mesh grids which were glow-discharged for 90 sec, then the samples were blotted and vitrificated by plunging into liquid ethane with a Vitrobot (FEI) operated at 4 °C and 100% humidity.

The frog erythrocyte nuclei were isolated from an erythrocyte suspension of *Rana catesbiana* (gift from Qin lab, Research centre for eco-environmental sciences). Erythrocytes were pelleted by centrifugation at 800 g and suspended in 110 mM PBS buffer (diluted by ddH_2_O). For lysis of the cytoplasmic membrane, erythrocytes were resuspended in 110 mM PBS buffer, containing 0.5% Nonidet P-40. The resuspension was then incubated at room temperature for 5 min. Nuclei were collected at 1000 g and washed twice and resuspended in 35 mM PBS buffer waiting for freezing. The frog erythrocyte nuclei were also chemically fixed by 0.5% glutaraldehyde and 1% paraformaldehyde. The nuclei were further cryo-protected by glycerol at a final concentration of 3%. Aliquots of 1.5 μl sample (~ 700 cells) were applied onto glow-discharged Quantifoil R2/1 300 mesh holey carbon grids, incubated for 10 s at 37 °C and 20% humidity, blotted for 8 s with a filter paper and then plunged into liquid ethane using an FEI EMGP (Thermo Fisher Sci).

### Cryo-FIB milling

Cryo-FIB milling were performed using Helios NanoLab 600i Dual Beam SEM (FEI, Netherlands) with a field emission electron source, gallium ion source and the in-lens electron detector. The frozen grids of nuclei were transferred with the cryo-transfer shuttle into the SEM chamber by using Quorum PP3000T cryo-transfer system (Quorum Technologies, East Sussex, UK) under −180 °C.

During the cryo-FIB milling process, the milling angle between the FIB and the specimen surface was set to 5–10°. The milling was performed parallel from two sides to produce vitrified cell lamella^39^. The accelerating voltage of the ion beam was kept at 30 kV, and the ion currents were in the range from 0.43 nA to 40 pA. The rough milling utilized a strong ion beam current of 0.43 nA and the final fine milling was operated with a small ion beam current of 40 pA. The thickness of the residual thin lamella with a good quality was <150 nm.

### Datasets

Five simulated tomogram datasets and six real tomograms were used to evaluate the performance of REST. We produced four simulated sub-tomograms datasets (SIM1-4) of containing one nucleosome particle generated from the PDB:3AFA and one simulated tomogram (SIM5) containing 64 nucleosome particles generated from the atom model of 177 bp nucleosome (see above for details). Real tomograms datasets were either downloaded from the EMPIAR (EMPIAR-10045, the purified ribosome dataset, EM1; EMPIAR-10499, ribosome dataset in situ from pneumococcus, EM6) or collected by ourselves (tomogram of the 147 bp recombinant nucleosome, EM2; tomogram of the mixed recombinant nucleosome core particle and nucleosomes with linker DNA, EM3; tomogram of the lamella of frog erythrocyte nuclei thinned by cryo-FIB, EM4, and mixed samples including ribosome and HBV particles, EM5). All images were recorded using SerialEM^40^. The detailed information of these datasets is summarized in Supplementary Table 2. The detailed information of training dataset used for model training is summarized in Supplementary Table 3.

### Detailed implementation in real nucleosome datasets

#### NMA of nucleosomes

For the NMA data processing of nucleosomes in this study, a series of atomic models were generated from nucleosomes with 147 bp DNA (PDB: 3AFA) by using a linear relationship between the amplitudes of normal modes 7 and 13. A gradual transition between the two ends, which represented a continuum of nucleosome conformations, was simulated. Equal random amplitudes uniformly distributed in the range [−250, 250] were used for the two normal modes 7 and 13. To visualize obvious continuous conformational changes, nucleosomes with long linker DNA were also studied. The atomic model of the 177 bp nucleosome was generated from PDB (7DBP) by removing the chain of H1. The following NMA was performed in a similar process as in the study of nucleosomes with 147 bp DNA.

### Training model for restoration

To restore the tomograms of real nucleosome data, we used the simulated data that mimicked the real collection conditions to train the model. The SNR of the simulated data was also ensured to be close to the real data. Specifically, the training data were deposited into cubic subvolumes of 64 voxels at a pixel spacing of 4.44 Å. The training pairs were normalized before training. The other steps are described above.

### Tomogram reconstruction

For the recombinant nucleosome datasets, the tilt movies were processed in Warp^41^, and the generated stacks were aligned using IMOD^42^. The reconstructions were generated from Warp using a pixel spacing of 4.44 Å, which was as same as that in the training dataset.

### Restoring the real tomograms

The tomograms were normalized before restoration. After training, the model was used to restore the tomogram reconstructed in Warp. The IsoNet strategy of prediction was implemented and used for restoration. We split the entire tomogram into small subvolumes in 64 voxels to predict them separately. Then, output 3D chunks were combined to produce the final output.

### Single-particle analysis of the mixed nucleosomes

We collected and processed the same mixed samples with the single-particle method. After 2D classification and 3D classification with Relion, we selected one class which had the feature of nucleosome with DNA. After refinement in Relion, a 3.7 Å map was obtained as the reference for REST-restored density.

### Subtomogram averaging of nucleosomes in frog erythrocyte nuclei

After the 3D tomogram reconstruction, an atomic structure of nucleosome core particle (PDB: 3AFA) was filtered to 60 Å and used as the template to determine the position of presumptive nucleosomes in the erythrocyte nuclei by template-matching in Warp. The determined nucleosome densities within the tomograms were extracted in Warp with a box size of 36 voxels (194 Å). These volumes were then projected into 2D slices (36 slices) and sorted into different classes through 2D classification in Relion. Particles in the good looking 2D classes were then subject to multi-round 3D classification in Relion. After the iterative 3D classification, classes with clear nucleosome features were selected and displayed (Fig. 6D and Supplementary Fig. 5). The particles in the class with clear feature of nucleosome with linker DNA were selected and subjected to further 3D classification. Six classes were finally achieved which showed the difference of linker DNA (Fig. 6D and Supplementary Fig. 5).

### Template matching in simulated tomograms

Since the ground truth of the real coordinates in the simulated tomograms has been already known, the calculated coordinate can be obtained through template matching in Dynamo^43^. Thus, by subtracting the real coordinates from the calculated coordinates in the X-, Y- and Z- directions, the shift of the corresponding particle coordinates could also be determined. At the same time, each particle returned a CC value during the calculation, and further comparison was made between the raw data and the result using REST.

### The 3D visualization

IMOD was used to visualize the 2D slices, and UCSF Chimaera^44^ and UCSF ChimeraX^45^ were used to visualize the 3D tomograms and subvolumes. Schematics were drawn using Adobe Illustrator.

### Reporting summary

Further information on research design is available in the Nature Portfolio Reporting Summary linked to this article.

## Supplementary information

Supplementary Information

Reporting Summary

## Data Availability

The data that support this study are available from the corresponding authors upon request. Datasets used in this study, including raw tomograms, restored tomogram and the trained model are deposited into the publicly available repository Figshare [10.6084/m9.figshare.22591465.v1]. Structural for training and comparisons were performed with 147 bp human nucleosome structure (PDB accession 3AFA), 177 bp human nucleosome structure (PDB accession 7DBP), *Trypanosoma brucei* ribosome (PDB accession 4V8M), *M. pneumoniae* 70 S ribosome (EMDB accession EMD-11999 (ref. ^28^.)) and HBV particle (EMDB accession EMD-20670 (ref. ^33^.)).

## Code availability

The code and tutorial are available at the GitHub repository [https://github.com/Zhang-hn1125/REST].
