# Supplementary material for: A method for restoring signals and revealing individual macromolecule states in cryo-ET, REST
Source: Nat Commun. 2023 May 22;14:2937. doi: 10.1038/s41467-023-38539-w (PMC10203133; doi:10.1038/s41467-023-38539-w)
Supplement: Supplementary file 1 — Supplementary Information [file 41467_2023_38539_MOESM1_ESM.pdf]

Supplementary Information for:

REST: A method for restoring signals and revealing individual macromolecule states in cryo-ET

Authors: Haonan Zhang, Yan Li, Yanan Liu, Dongyu Li, Lin Wang, Kai Song, Keyan Bao, Ping Zhu

Inventory of Supporting Information: Supplementary Figures and Tables

Supplementary Fig. 1 .... **2**

Supplementary Fig. 2 .... **3**

Supplementary Fig. 3 .... **4**

Supplementary Fig. 4 .... **5**

Supplementary Fig. 5 .... **6**

Supplementary Fig. 6 .... **7**

Supplementary Fig. 7 .... **8**

Supplementary Fig. 8 .... **9**

Supplementary Table 1 .... **10**

Supplementary Table 2 .... **11**

Supplementary Table 3 .... **12**

Supplementary Table 4 .... **13**

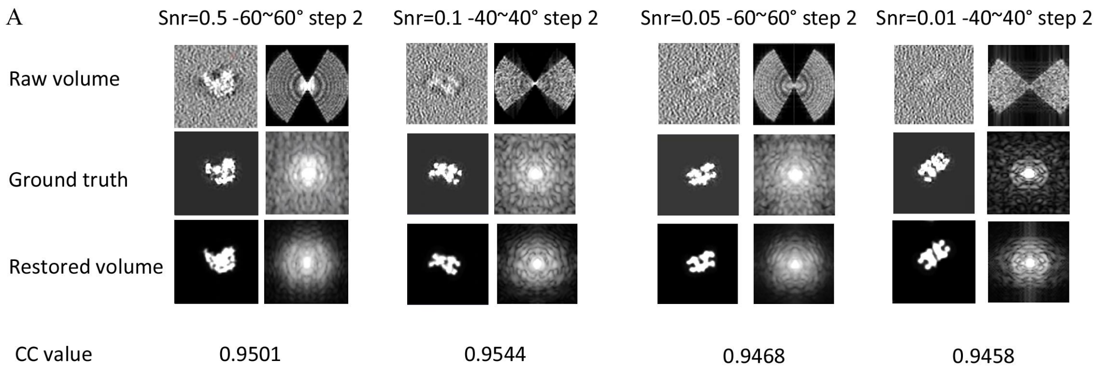

Supplementary Fig. 1. The performance of REST restoration under different simulation conditions. The volumes of particles with different noise and missing wedge information superimposed (raw volume), the generated high-quality particles (ground truth), and the REST restored particles (restored volume) are shown in the top, middle and bottom rows respectively. The left side of each pair is the XY-slice view, and the right side is the Fourier transform of the left particles. The CC value represents the cross coefficient between the restored volume and ground truth.

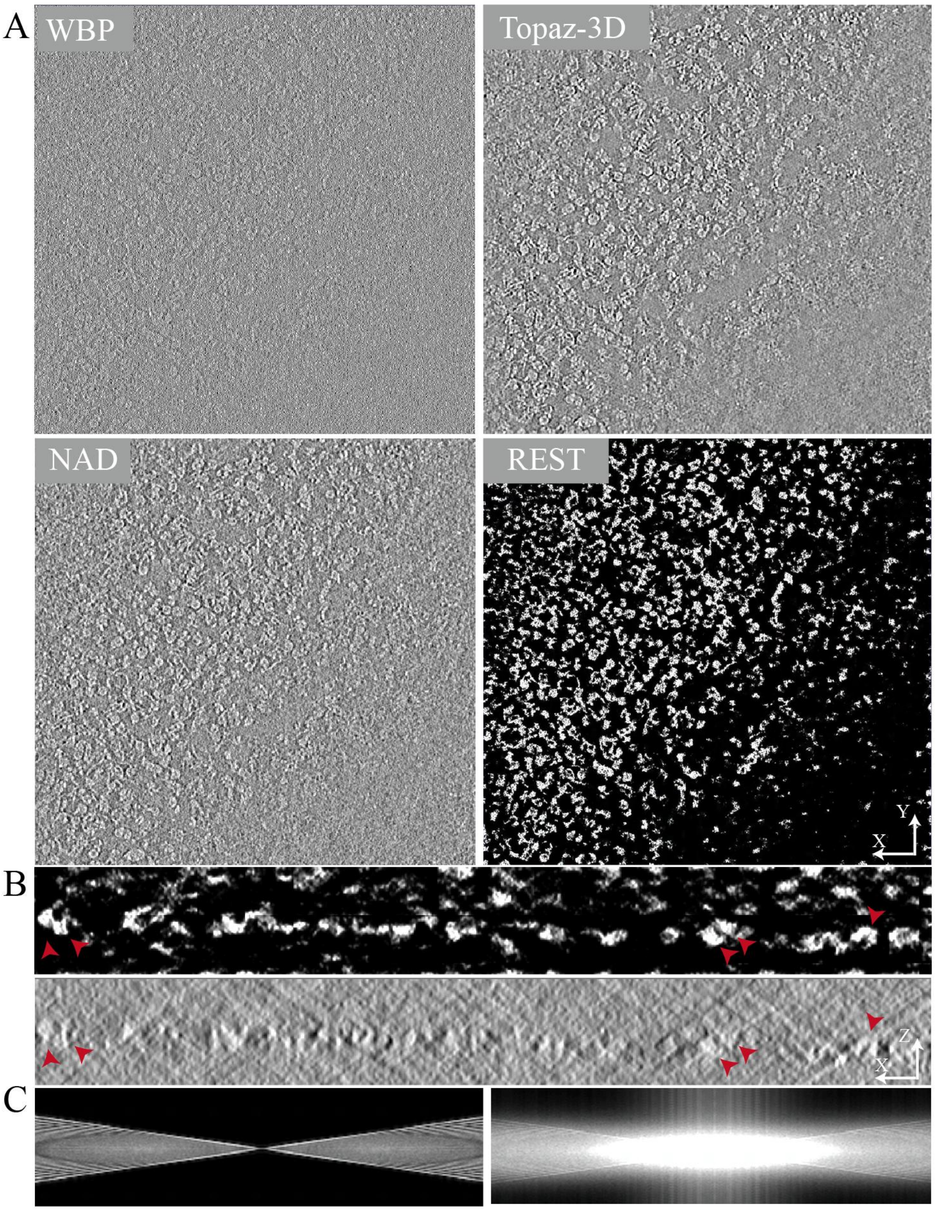

Supplementary Fig. 2. Enhancement of the visualization of nucleosomes (EM2) in tomograms using REST. (A) Compared with the raw data, the NAD and Topaz-Denoise, REST methods can be used to thoroughly remove the noise shown in the tomographic slices. **(B)** The XZ-slice views of the REST-restored tomogram (top) and the denoised tomogram using Topaz-Denoise (bottom). **(C)** Fourier transforms of the raw tomogram (left) and REST-restored tomogram (right).

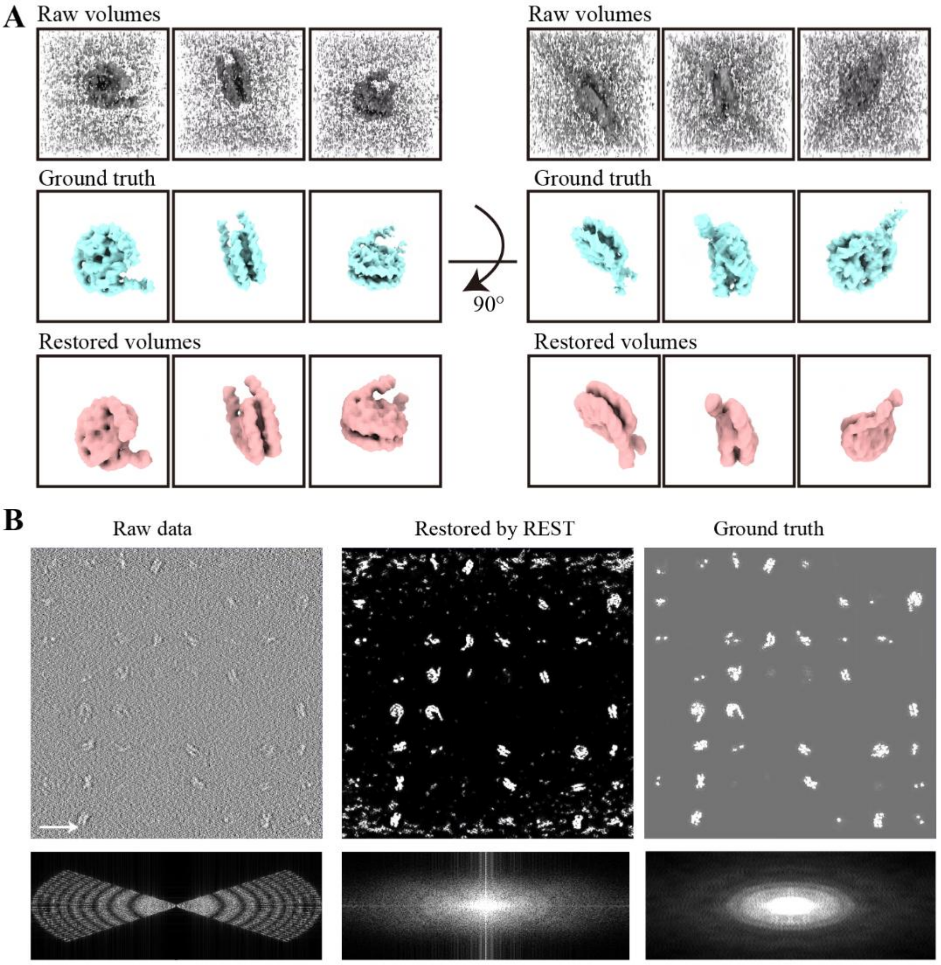

Supplementary Fig. 3. (A) The 3D volume of input particles (raw volumes) (top), the corresponding ground truth (middle) and the restored volumes using REST (bottom). **(B)** Top: The XY-slice views of the simulated raw tomogram (left), REST-restored tomogram (middle) and the ground truth; Bottom: the corresponding Fourier transforms of the above tomograms.

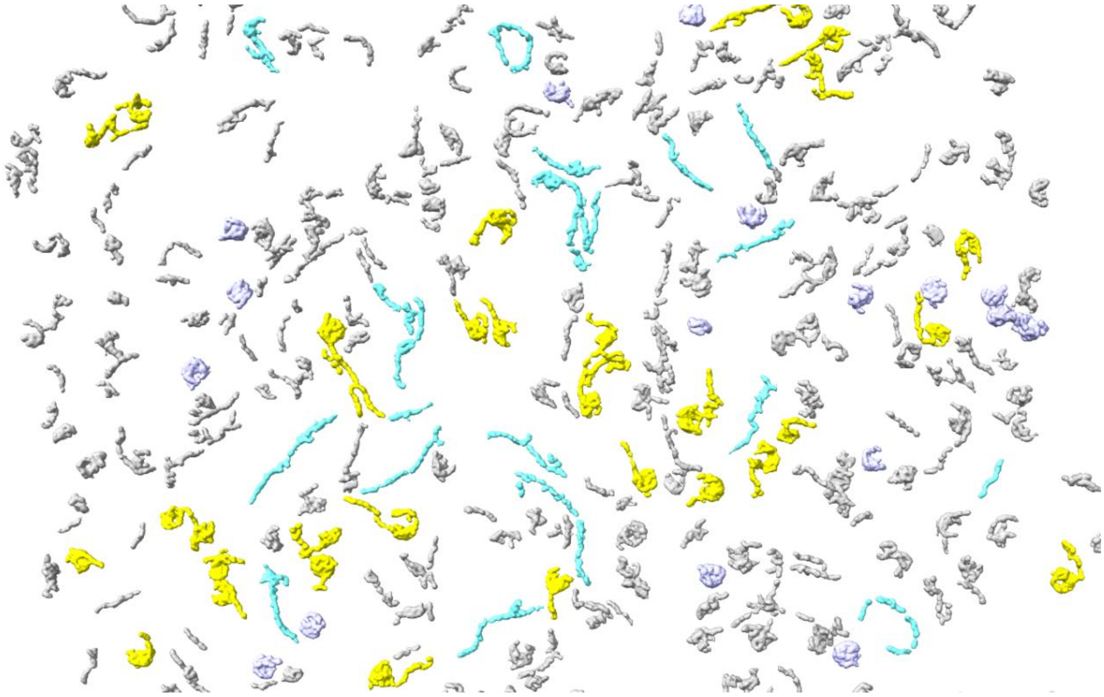

Supplementary Fig. 4. The whole tomogram of reconstituted nucleosomes restored by REST. A portion of nucleosome core particles, nucleosomes with linker DNA and free DNA are shown in purple, yellow and cyan, respectively.

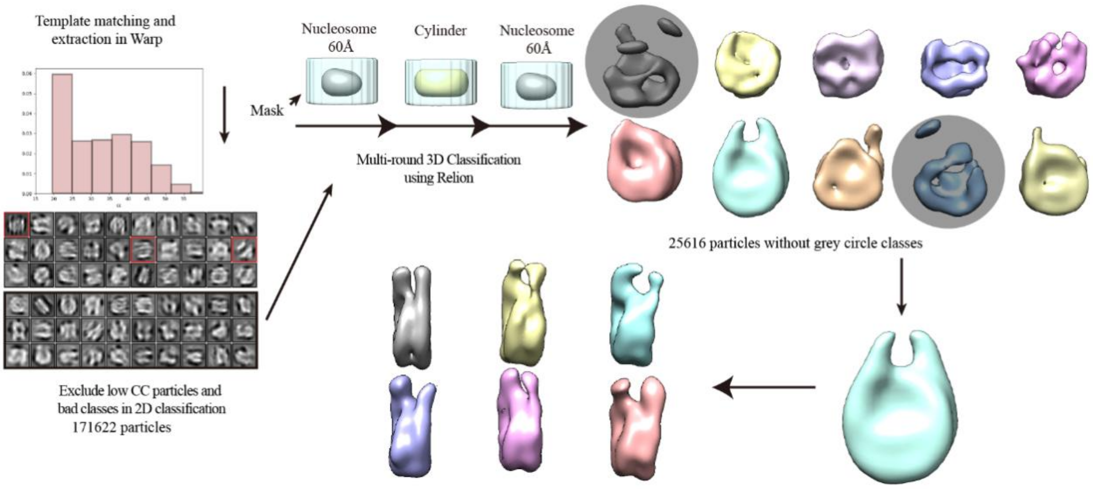

Supplementary Fig. 5. Subtomogram averaging workflow of nucleosome structure from frog erythrocyte nuclei.

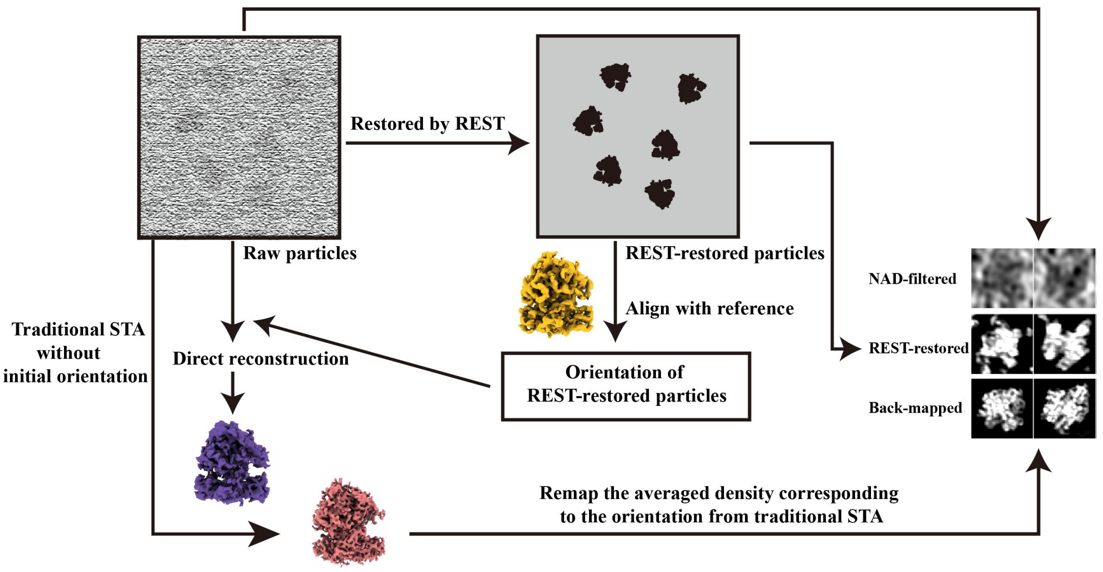

Supplementary Fig. 6. The flow chart shows that REST assists in the orientation determination in STA.

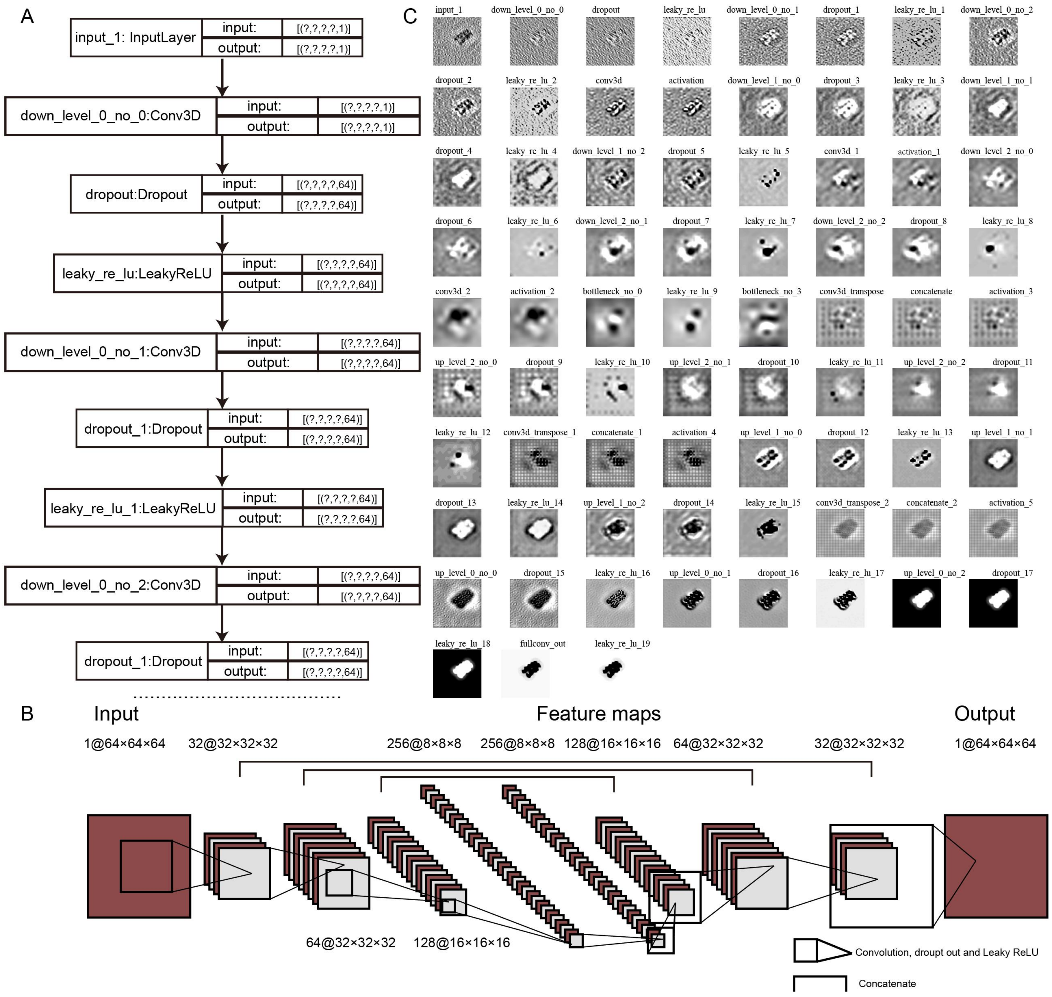

Supplementary Fig. 7. The process of restoration by the REST-trained model. **(A)** The example layers of neural network framework in REST. **(B)** The architecture of neural network in REST. **(C)** The slice of the feature map of each layer of the model when restoring(predicting) the nucleosome data.

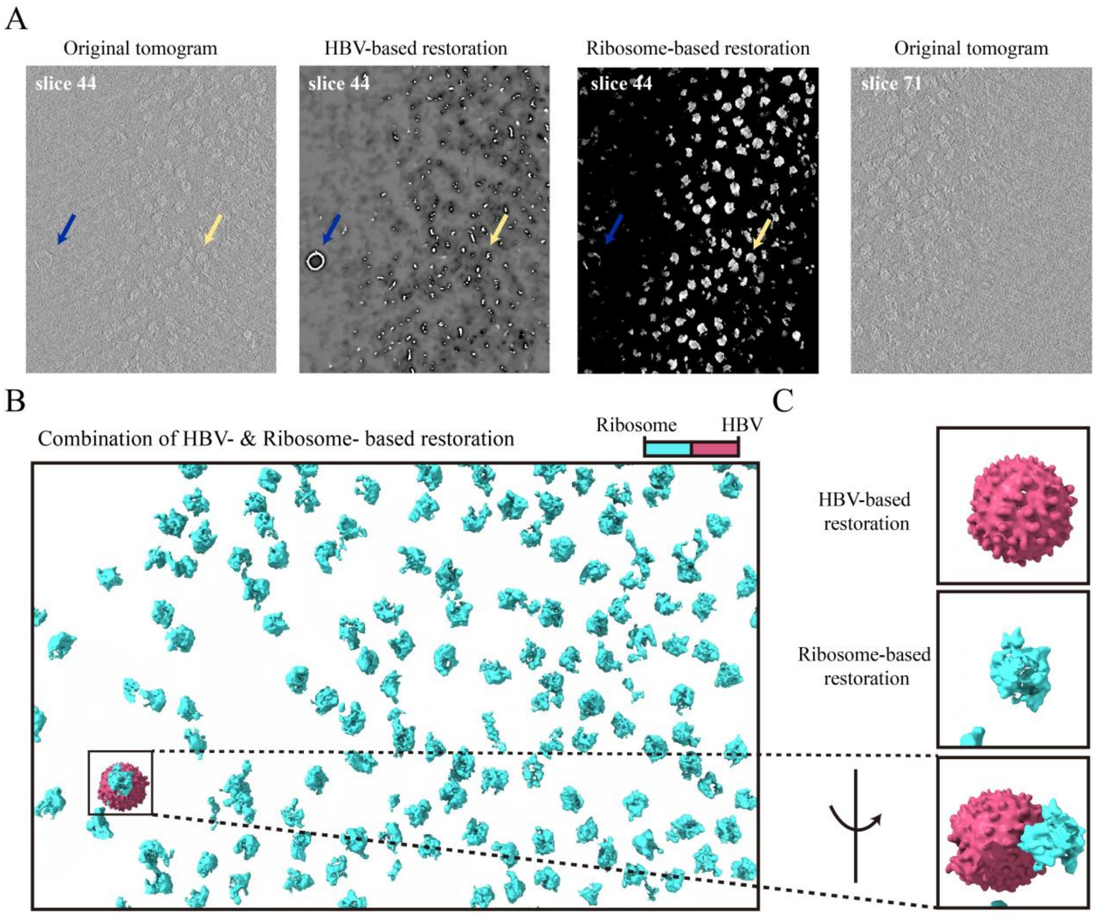

Supplementary Fig. 8. REST could specifically restore target molecules of interest. The REST restoration of HBV/ribosome mixed sample. **(A)** The left 3 panels: a slice (slice 44) of the original tomogram (left) and the corresponding slice of REST restored tomogram based on HBV-strucutre model (middle) and ribosome-structure model (right) respectively. For comparison, another slice (slice 71) of the original tomogram is also shown. **(B)** A combined 3D visualization of the REST restored tomograms based on HBV-model (pink) and ribosome-model (cyan). **(C)** Enlarged view of the black box in (B) showing the HBV-based restoration (top) and the ribosome-based restoration (middle) respectively, and the combined view of the boxed area from another viewing angle (bottom).

Supplementary Table 1: Comparison of denoising methods based on the estimated SNR (in dB)

SNR	Dataset	
Method	EM1 (ribosome)	EM2 (nucleosome)
Raw data	0.352	0.05
Gauss filter	0.419	—
Wiener filter	0.598	—
NAD	0.652	0.14
Topaz-Denoise	0.815	0.23
REST	7.337	9.57

Supplementary Table 2: Detailed information of the simulated and real datasets.

Details in data collection									
	SIM1	SIM2	SIM3	SIM4	SIM5	EM2	EM3	EM4	EM5
Pixel size (Å)	4.44	4.44	4.44	4.44	4.44	1.36	1.36	2.73	1.36
Defocus(μm)	-3.5	-3.5	-3.5	-3.5	-3.5	-4	-5	-5	-5
Voltage (kV)	300	300	300	300	300	300	300	300	300
SNR	0.5	0.1	0.05	0.01	0.05	—	—	—	—
Angle, step (°)	±60, 2	±40, 2	±60, 2	±40, 2	±40, 5	±50, 3	±55, 3	±55, 3	±50, 3
Specimen	3AFA	3AFA	3AFA	3AFA	177 bp*	Core particle*	Mixed*	Nuclei*	Ribosome&HBV

*Nucleosome sample.

Supplementary Table 3: Detailed information of training data used for training model.

Detailed parameters of training dataset											
	EM1	SIM1	SIM2	SIM3	SIM4	SIM5	EM2	EM3	EM4	EM5	EM6
Pixel size (Å)	4.34	4.44	4.44	4.44	4.44	4.44	4.44	4.44	4.44	6.8	6.8
Defocus(μm)	-	-3.5	-3.5	-3.5	-3.5	-3.5	-4	-5	-5	-5	-5
Voltage (kV)	-	300	300	300	300	300	300	300	300	300	300
SNR	-	0.5	0.1	0.05	0.01	0.05	0.05	0.05	0.02	0.01	0.01
Angle, step (°)	-	±60, 2	±40, 2	±60, 2	±40, 2	±40, 5	±50, 3	±55, 3	±55, 3	±50, 3	±60, 3
Strategy	1	2	2	2	2	2	2	2	2	2	2
Sample size	2578	3000	3000	3000	3000	3000	3000	3000	3000	3000	3000
Ground truth	Averaged ribosome	3AFA	3AFA	3AFA	3AFA	177 bp*	Core particle*	Mixed*	Nuclei*	Ribosome&HBV	EMD-11999

Supplementary Table 4: Cross coefficient (CC) between the T-I volume and restored volume shown in Fig. 5.

	Particle type	
	Nucleosome core particles	Nucleosomes with linker DNA
Particle 1	0.81	0.64
Particle 2	0.84	0.89
Particle 3	0.78	0.72
Particle 4	0.77	0.76
Particle 5	0.78	0.57
Particle 6	0.70	0.72
