# Supplementary material for: A method for restoring signals and revealing individual macromolecule states in cryo-ET, REST
Source: Nat Commun. 2023 May 22;14:2937. doi: 10.1038/s41467-023-38539-w (PMC10203133; doi:10.1038/s41467-023-38539-w)
Supplement: Supplementary file 2 — Reporting Summary [file 41467_2023_38539_MOESM2_ESM.pdf]

nature research

Corresponding author(s): Ping Zhu

Last updated by author(s): Apr 15, 2023

Reporting Summary

Nature Research wishes to improve the reproducibility of the work that we publish. This form provides structure for consistency and transparency in reporting. For further information on Nature Research policies, see our Editorial Policies and the Editorial Policy Checklist.

Statistics

For all statistical analyses, confirm that the following items are present in the figure legend, table legend, main text, or Methods section.

n/a	Confirmed	
☐	☒	The exact sample size (*n*) for each experimental group/condition, given as a discrete number and unit of measurement
☐	☒	A statement on whether measurements were taken from distinct samples or whether the same sample was measured repeatedly
☒	☐	The statistical test(s) used AND whether they are one- or two-sided<br>*Only common tests should be described solely by name; describe more complex techniques in the Methods section.*
☐	☒	A description of all covariates tested
☐	☒	A description of any assumptions or corrections, such as tests of normality and adjustment for multiple comparisons
☒	☐	A full description of the statistical parameters including central tendency (e.g. means) or other basic estimates (e.g. regression coefficient) AND variation (e.g. standard deviation) or associated estimates of uncertainty (e.g. confidence intervals)
☒	☐	For null hypothesis testing, the test statistic (e.g. F, t, r) with confidence intervals, effect sizes, degrees of freedom and P value noted<br>*Give P values as exact values whenever suitable.*
☐	☒	For Bayesian analysis, information on the choice of priors and Markov chain Monte Carlo settings
☐	☒	For hierarchical and complex designs, identification of the appropriate level for tests and full reporting of outcomes
☒	☐	Estimates of effect sizes (e.g. Cohen's d, Pearson's r), indicating how they were calculated

Our web collection on statistics for biologists contains articles on many of the points above.

Software and code

Policy information about availability of computer code

Data collection	The Cryo-EM data was collected using software SerialEM3 .7.
Data analysis	The software for structure determination and illustration includes Warp 1.0.9, Relion 3.0, HEMNMA_3D, EMAN2.9, Xmipp3, Dynamo1.1.332, UCSF Chimera 1.16, UCSF ChimeraX 1.3, IMOD4.8.49, and ourselves code are available at https://github.com/Zhang-hn1125/REST.

For manuscripts utilizing custom algorithms or software that are central to the research but not yet described in published literature, software must be made available to editors and reviewers. We strongly encourage code deposition in a community repository (e.g. GitHub). See the Nature Research guidelines for submitting code & software for further information.

Data

Policy information about availability of data

All manuscripts must include a data availability statement. This statement should provide the following information, where applicable:
- Accession codes, unique identifiers, or web links for publicly available datasets
- A list of figures that have associated raw data
- A description of any restrictions on data availability

The data that support this study are available from the corresponding authors upon reasonable request. Datasets used in this study, including raw tomograms, restored tomogram, the trained model and the tutorial datasets are deposited into the publicly available repository Figshare (https://doi.org/10.6084/m9.figshare.22591465.v1).
Structural for training and comparisons were performed with 147bp human nucleosome structure (PDB accession 3AFA (http://dx.doi.org/10.2210/pdb3afa/pdb)), 177bp human nucleosome structure (PDB accession 7DBP (http://doi.org/10.2210/pdb7DBP/pdb)), Trypanosoma brucei ribosome (PDB accession 4V8M (http://doi.org/10.2210/pdb4V8M/pdb)), M. pneumoniae 70S ribosome (EMDB accession EMD-11999 and HBV particle (EMDB accession EMD-20670).

Field-specific reporting

Please select the one below that is the best fit for your research. If you are not sure, read the appropriate sections before making your selection.

[x] Life sciences   [ ] Behavioural & social sciences   [ ] Ecological, evolutionary & environmental sciences

For a reference copy of the document with all sections, see nature.com/documents/nr-reporting-summary-flat.pdf

Life sciences study design

All studies must disclose on these points even when the disclosure is negative.

Sample size	Cryo-EM structures and tomograms were processed from the best data set after extensive optimization of sample prep and data process.
Data exclusions	No data were excluded.
Replication	We use the deep-learning method to repeat the training process at least 3 independent times to get the same result, and apply the model multiple times to get the same result.
Randomization	Our data does not require random sampling during processing
Blinding	No blinding experiments are included. An individual person conducted each experiment according to their expertise.

Reporting for specific materials, systems and methods

We require information from authors about some types of materials, experimental systems and methods used in many studies. Here, indicate whether each material, system or method listed is relevant to your study. If you are not sure if a list item applies to your research, read the appropriate section before selecting a response.

Materials & experimental systems

n/a	Involved in the study
[x]	[ ] Antibodies
[x]	[ ] Eukaryotic cell lines
[x]	[ ] Palaeontology and archaeology
[x]	[ ] Animals and other organisms
[x]	[ ] Human research participants
[x]	[ ] Clinical data
[x]	[ ] Dual use research of concern

Methods

n/a	Involved in the study
[x]	[ ] ChIP-seq
[x]	[ ] Flow cytometry
[x]	[ ] MRI-based neuroimaging
